# Lumbar Spine Fusion Patients’ Use of an Internet Support Group: Mixed Methods Study

**DOI:** 10.2196/jmir.9805

**Published:** 2019-07-04

**Authors:** Janni Strøm, Mette Terp Høybye, Malene Laursen, Lene Bastrup Jørgensen, Claus Vinther Nielsen

**Affiliations:** 1 Elective Surgery Centre Silkeborg Regional Hospital Silkeborg Denmark; 2 Section for Clinical Social Medicine and Rehabilitation Department of Public Health Aarhus University Aarhus Denmark; 3 Interacting Minds Centre Department of Clinical Medicine Aarhus University Aarhus Denmark; 4 Section for Public Health Department of Clinical Medicine Aarhus University Aarhus Denmark; 5 DEFACTUM Central Denmark Region Aarhus Denmark; 6 Regional Hospital West Jutland Central Denmark Region Aarhus Denmark

**Keywords:** spinal fusion, medical informatics, self-help groups, eHealth, online social networking, social support

## Abstract

**Background:**

Internet use within health care contexts offers the possibility to provide both health information and peer support. Internet Support Groups (ISGs) for patients may offer advantages, which are not found in face-to-face support. In patients undergoing lumbar spine fusion (LSF), ISGs could have a particular potential, as peer support on the web might bridge the decreased satisfaction with social life and social isolation found within these patients. ISGs might in this way contribute to increasing the functioning and overall health-related quality of life. However, LSF patients may generally belong to a group of citizens not prone to internet and online peer support. However, our knowledge of how LSF patients use ISGs is limited.

**Objective:**

The aim of this study was to describe the characteristics of users of an ISG and thematically explore the content of ISG interactions in Danish patients undergoing instrumented LSF because of degenerative spine disorders.

**Methods:**

Participants were recruited from a randomized controlled trial and included in a prospective cohort with a mixed methods design. Sociodemographic characteristics and information on psychological well-being (symptoms of anxiety and depression) were obtained at baseline and 1 to 5 weeks before surgery. Usage of the ISG was registered from baseline until 3 months after surgery. All posts and comments were collected, and content analysis was performed.

**Results:**

A total of 48 participants comprised the study population, with a mean age of 53 years (range 29-77). Of the participants, 54% (26/48) were female, 85% (41/48) were cohabitating, 69% (33/48) were unemployed, and the majority (69% [33/48]) had secondary education. Approximately one-third of the participants had symptoms of depression (35%, 17/48) and anxiety (29%, 14/48). Overall, 90% (43/48) of the participants accessed the ISG. No correlations were found between sociodemographic characteristics and access to the ISG. Women were more prone to be active users, contributing with posts (*P*=.04). Finally, active users contributing with posts or comments had viewed more pages, whereas passive users, users without posts or comments, had more interactions with the ISG (*P*<.001). The ISG contained 180 conversation threads, generating 354 comments. The 180 conversation threads in the ISG were constituted by 671 independent dialogue sequences. On the basis of those 671 dialogue sequences, 7 thematic categories emerged.

**Conclusions:**

Sociodemographic characteristics were not predictors of ISG use in this study, and active use was found to be gender dependent. Content of interactions on the ISG emerged within 7 thematic categories and focused on social recognition, experience of pain or use of pain medication, experience of physical activity or physical rehabilitation, expression of psychosocial well-being, advising on and exploring the ISG, and employment, which seemed to correspond well with the prevalent occurrence of symptoms of anxiety and depression.

## Introduction

### Background

The aim of this study was to describe the characteristics of users of an Internet Support Group (ISG) and thematically explore the content of interactions in an ISG for patients undergoing instrumented lumbar spine fusion (LSF). The ISG was embedded on a platform also containing animated information and training instructions, designed to support LSF patients primarily within the first 3 months after LSF.

Peer support has been found to be applicable in the general population [1], and the desire from patients to connect with each other has been found to grow [2]. During the last decade, the body of literature exploring the use of ISG in cancer patients, patients with depression, patients with HIV/AIDS or other long-term conditions has grown [3-9]. Some of these studies find a positive effect, when providing ISG, on patients’ depression, anxiety, or on quality of life [6-9], and it has been suggested that connecting with others on an ISG reduces the sense of isolation [10,11]. Other studies have mixed results or no positive results [12-14]. In addition, some studies even find negative results, as a Web-based survey suggests that the lack of actual physical proximity makes relationships developed within an ISG less meaningful and actually makes patients feel even more isolated [4].

The possibility of providing electronic health (eHealth) interventions is growing with the increasing availability of the internet [15,16-19]. With the use of ISG, peer support is made easier for citizens who, because of their health status, cannot participate in face-to-face groups, and peer support is made easier for citizens in remote areas, citizens with social anxiety, or those who feel uncomfortable disclosing personal experience in a room with others [9,20]. Particularly for patients with anxiety and depression, the ability to remain anonymous might enable them to use ISGs and gain support from peers [21].

In this study, LSF patients were targeted. These patients represent a quite substantial group of back patients, as fusion of the lumbar spine is a commonly performed surgical procedure when treating various conditions of the spine [22]. Approximately 488,000 spinal fusions were performed during US hospital stays in 2011 (3.1% of all operating room procedures) [23], lumbar fusions being the most common type of fusion performed, approximately 210,000 operations in the United States each year [22].

Introducing ISGs to patients undergoing LSF might have potential, as anxious and depressed patients are found to be more prone to take part in an ISG [24], and as symptoms of anxiety and depression are found in approximately one-third of these patients [25-28]. Furthermore, limiting seated transportation is recommended during the first 6 weeks after surgery, and active rehabilitation starts no sooner than 3 months after surgery [29]. This limits LSF patients’ ability to attend face-to-face meetings in this period, and studies find a significant decline in satisfaction with social life after LSF [30], predicting worse score in health-related quality of life after surgery [31]. Thus, several LSF patients are found to have symptoms of anxiety and depression and to be bound to their homes, and thus they might be more prone to use an ISG. In a previously published randomized controlled trial (RCT), we examined the effect of the total Web-based platform (including animated information and training instructions, a click-through diary, written information, and the ISG) on symptoms of anxiety and depression and on pain. We found no additional effect of the Web-based platform on any of the outcome parameters [14].

### Objective

We need to further explore the use of an ISG within this group of patients. Higher age and lower socioeconomic background seem to characterize people who are less frequent internet users and who potentially benefit less from Web-based support [6,32-35], characteristics which generally match those of LSF patients [36]. Thus, we need to explore and describe the characteristics of users and describe the content of the Web-based interaction in an ISG provided to LSF patients, to inform future design and use of such interventions to this patient population. In addition, most studies exploring the use of ISG use already established forums, and little is known about newly initiated forums [37].

## Methods

### Study Design and Setting

This study’s population comprises the patients enrolled and randomized from September 2015 to May 2017 [14] to an intervention group in a 2-arm RCT at a single-center orthopedic spine department in Denmark.

The population comprises 48 participants receiving access to an ISG as part of their pre and postsurgical care. All patients were scheduled for first time 1- to 3-level instrumented LSF because of degenerative disorders. Patients were excluded if they were below the age of 18, if they had a known psychotic disorder, if they were unable to communicate in Danish, or had no access to the internet. Patients were invited to participate in the study by a study nurse, receiving written and verbal information, when the patients attended the outpatient clinic and were scheduled for surgery.

Final decision of participation was made, and written consent was collected at a following baseline visit 1 to 5 weeks before surgery. At baseline, participants were introduced and received access to the ISG, and a 15-min hands-on introduction in the use of the website was provided by a study nurse. During the introduction, all participants were encouraged to share their experience, thoughts, and questions on the ISG, and all were encouraged to use a respectful tone. Participants were told to use the ISG in a way that made sense to them and fulfilled their needs. During the study, the participants received no notifications of activity on the ISG. Posts or comments would only be visible when the participants choose to access the ISG.

Patients were continuously included as they attended the outpatient clinic, but because of a variation of patient flow, the speed of inclusion was uncertain. Thus, 6 former patients were invited as facilitators to start the dialogue and post updates in the common space, creating activity and engaging the first participants entering the group. These 6 facilitators were not excluded from the ISG during the study period, and 1 of them contributed to the dialogues throughout the study. We did not include these former patients in the descriptive analysis, describing the characteristics of users during the first 3 months after surgery. The main reason for this was that they had a different role than those participating in the study, as they were committed differently. However, their comments and posts are included in the content analysis, as these were part of the conversational context of what was posted by study participants. The content data were collected from the first participant through to 6-month follow-up of the last attending participant.

It was decided to promote implementation by providing participants with easy access. This was decided as a previous study introducing a similar Web-based platform for patients undergoing total hip replacement found the access rate as low as 61% [38]. With reference to theories within implementation [39], attention was given toward easy access. Thus, patients who were not in possession of a tablet were offered the use of one from baseline and until 3 months after surgery.

The science ethics committee was notified of the study, and it did not find that permission was required (J.no. 1-10-72-36-15). Data management and security were approved by the Danish Data protection agency (J.no. 2014-41-3583). In line with the Helsinki Declaration [40], patients were informed about the study both in writing and verbally and had at least 24 hours to consider their participation.

### The Internet Support Group

The ISG could be accessed from any browser through a designated website. The ISG was closed to the public, and participants logged on using an individual password. Participants were assured that all data were kept for research purposes only and that all correspondence was kept in accordance with data management and security regulations. Furthermore, all participants were informed that it would not be possible to identify any of the participants in any published work. A researcher and a study nurse provided technical support if this was needed, and the researcher would intervene if any offensive remarks were posted or if an aggressive tone was used. No technical support or mediation of such behavior was ever needed, and no intervention or moderation was provided by either the researcher or the study nurse.

**Figure 1:**
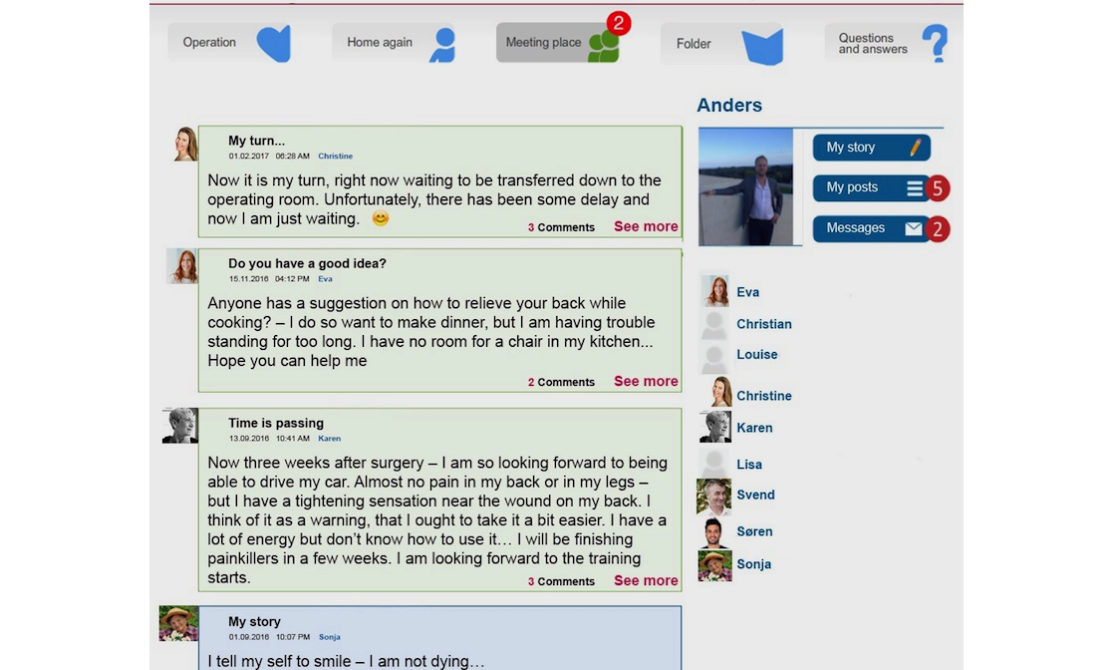
The message board visible to all lumbar spine fusion patients assigned to an Internet Support Group. The board is a reconstruction, names are invented, pictures are stock photos, and the text is fictional, based on inspiration from real posts.

**Figure 2:**
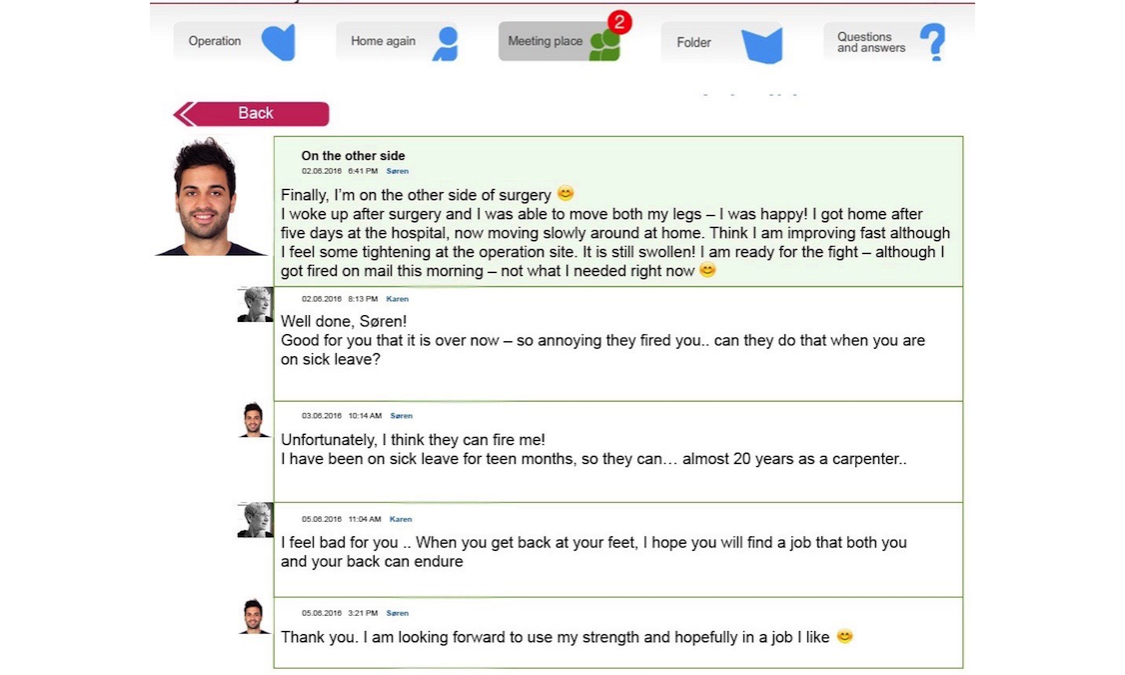
The message board with comments, visible to all lumbar spine fusion patients assigned to an Internet Support Group. This message board is a reconstruction, names are invented, pictures are stock photos, and the text is fictional, based on inspiration from real posts.

**Figure 3:**
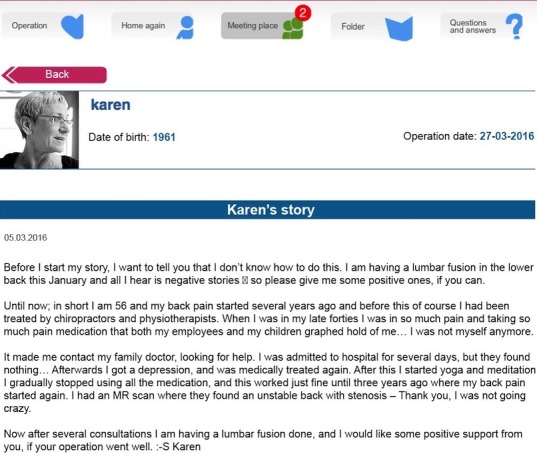
Personal page on the Internet Support Group platform, with the possibility to upload a picture, note date of birth and surgery, and write a personal background story. The page is a reconstruction, the name and dates are invented, picture is a stock photo, and the text is fictional based on inspiration from a real story.

The ISG comprised a message board visible to all participants (Figures 1 and 2). In this space, participants could post their experience, thoughts, or questions for other participants to comment on. On a designated page, each participant could upload a picture, note date of birth and date of surgery, and write a personal or background story. No restrictions were made on as to what this personal or background story should include, and no restrictions were made on the length of the post or how frequently participants could write in this space (Figure 3). Everything written here was visible for all participants.

### Data Collection

Data collection to describe the characteristics of the use of the ISG during the first 3 months after surgery was partly done in person by a study nurse at baseline visit and partly by manually tracking activity on the ISG until the participant had passed 3 months after surgery. Baseline data were collected by a study nurse, and the data comprised gender, age, sociodemographic background, and psychological well-being. Marital status was classified as married/cohabiting or living alone (including widow, single, or divorced). Educational status was classified into 3 categories, using the International Standard Classification of Education 2011 [41]: basic education (early childhood education, primary education, and lower secondary education), secondary education (upper secondary education), and higher education (postsecondary nontertiary education, short-cycle tertiary education, bachelor’s or equivalent, master’s or equivalent, and doctoral or equivalent level). Employment status was classified as 1 of 3 categories: (1) employed/full or part-time, (2) pensioner/other (includes participants not employed for other reasons than illness or unemployment, such as housewife, on leave, or student), and (3) sick leave/unemployed. Psychological well-being was obtained using self-administered, paper and pencil Hospital Anxiety and Depression Scale (HADS), which was distributed in person. HADS is a 14-item scale, in 2 subscales for anxiety and depression. The cut-off point has been identified at a score of 8/21 for symptoms of anxiety and depression [42]. For anxiety, this gave a specificity of 0.78 and a sensitivity of 0.9. For depression, this gave a specificity of 0.79 and a sensitivity of 0.83 [42].

### Data Collection to Analyze the Content of Interactions

From baseline until 3 months after surgery, participants’ activity on the ISG was monitored manually. This was done by tracking the use of google analytics, which is a free Web analytics tool generating detailed statistics concerning user behavior on websites. Google analytics were used, including location data, browser data, device type, event type, and event time; user-generated content data, including all posts, comments and stories by individual users, and personal data, including location, access date, date of operation, and from which device the participant gained access.

Activity was measured as interactions; 1 interaction is when 1 participant interacts with the ISG. An interaction often comprised a group of page views taking place within the same session. Distinctions were made between posts and comments. A post is a new upload of a question, update, or other, and a comment is an answer or comments written in a thread of an uploaded post.

Participants accessing the ISG without making posts or comments are defined as passive users, and those participants who contribute with posts or comments are defined as active users. The entire group entering the ISG will be referred to as users. Time spent on pages was not utilized, as it was not possible to ascertain whether the ISG was used when the pages were open.

### Statistical Analysis

All data were coded to compute the data statistically and then entered twice into Excel. The data were then transferred to StataCorp. 2017. STATA Statistical Software: Release 15, here all statistical analyses were performed.

Demographics were utilized to describe the sample. Descriptive statistics were done using frequencies and percentages to describe the sample profile and summarize data. Means and standard deviations were reported for continuous variables. Nonparametric data were analyzed using Spearman correlation tests to detect correlation among variables. Kruskal-Wallis ranks tests and Wilcoxon rank-sum test were used to establish any significant differences among unordered groups.

### Content Analysis

Qualitative data, comprising posts and comments from the ISG in a 2-year period (from first patient entered until the last patient attended 6-month follow-up), were collected and analyzed to explore the content of interactions on the ISG. All posts and comments were collected manually from the ISG and managed using NVivo qualitative data analysis software; QSR International Pty Ltd. Version 12, 2018. Data were analyzed using a qualitative content analysis [43]. Taking an overall inductive approach, all correspondence was read through to get an impression of the general context. It was then organized using cross-sectional indexing [44], on the basis of similarities and variations in the dialogue among participants, to produce an overview of the thematic indicators and analytical categories of use of the ISG. On the basis of these inductive categories, an explanatory synthesis was aggregated from the categories to condense the impression of interactions, presented as individual thematic categories below.

## Results

### The Characteristics of Internet Support Group usage

This paper represents 48 patients undergoing instrumented LSF, and out of these participants, 57% of the participants were females. The mean age was 53 years (range 29-77). A total of 69% of the participants completed secondary education, whereas 27% of the participants had completed basic education, and only 6% of the participants had completed higher education. Preoperatively, a total of 40% of the participants were on sick leave or for other reasons unemployed; 31% of the participants were employed, and 29% of the participants were pensioners/other. Thus, approximately 70% of the participants were outside the labor market. A clear majority of participants were married/cohabitating (84%; Table 1).

**Table 1:** Sociodemographic characteristics of the participants (n=48).

Characteristics		Values
**Gender, n (%)**		
	Male	22 (46)
	Female	26 (54)
Age (years), mean age (range)		53 (29-77)
**Marital status, n (%)**		
	Married/cohabitating	41 (84)
	Living alone^a^	7 (15)
**Education, n (%)**		
	Basic education^b^	12 (25)
	Secondary education^c^	33 (69)
	Higher education^d^	3 (6)
**Employment status, n (%)**		
	Employed full/part time	15 (31)
	Pension/other^e^	14 (29)
	Sick leave/unemployed	19 (40)

^a^Includes participants who are widowed, single, or divorced.

^b^Basic education level comprised International Standard Classification of Education levels 0-2.

^c^Secondary education comprised International Standard Classification of Education levels 3.

^d^Higher education level comprised International Standard Classification of Education levels 4-8 [41].

^e^Includes participants not employed for reasons other than illness or unemployment, such as housewives, those on leave, or students.

A total of 59% of the participants (n=29) chose to borrow a tablet. No correlations were found among gender, age, or sociodemographic data or among any of the valuables of use in relation to whether the participant chose to borrow a tablet or not (*P*>.45).

The 48 participants had a total of 933 interactions on the ISG during their first 3 months after surgery. A total of 90% of the participants (n=43) were users of the ISG, and 5 participants (10%) never used the ISG. The mean number of interactions for the 48 participants was 19.4 (range 0-90, SD 19.28); however, the 3 most active users had a total of 25.5% (238) of all interactions.

In the 933 interactions, the total number of page views was 2093. The mean number of page views for each of the 48 participants was 42.7 (range 0-312, SD 62.84). Of the 48 participants, a total of 48% participants (n=23) participated with posts or comments. The total number of posts and comments the during first 3 months after surgery was 288, and the mean number of posts or comments for the 48 participants was 6 (range 0-61, SD 13). Spearman rho was performed, looking for correlation between variables of use of the ISG and age and education. No significant correlations were found (Table 2). Analysis was performed to detect differences between groups (Tables 3 and 4). No differences were found except for gender, indicating that contributing posts were more common in women.

At baseline, 14 participants (29%) scored 8 or more on the HADS anxiety subscale, indicating the presence of symptoms of anxiety, and 17 participants (35%) scored 8 or more on the HADS depression subscale, indicating the presence of symptoms of depression. A total of 8 (16%) of the participants had symptoms of both depression and anxiety, leaving only 25 participants (52%) without either symptoms of anxiety or depression (Table 5).

No significant differences were found among groups regarding the use of the ISG and the presence or absence of anxiety and depression (Table 6). However, participants with symptoms of anxiety tended to be more prone to contribute with posts or comments on the ISG than those without anxiety.

**Table 2:** Correlation between variables of use and demographic data. No significant correlations were found between age or education and the variables of Internet Support Group use (Spearman correlation).

Demographic data	Interactions		Page views		Posts		Comments	
	Spearman rho	*P* value	Spearman rho	*P* value	Spearman rho	*P* value	Spearman rho	*P* value
Age	–0.0706	.63	0.0530	.72	–0.1305	.37	–0.1110	.45
Education	–0.0920	.53	–0.1702	.24	0.0220	.88	–0.1076	.46

**Table 3:** Correlation between activity on the Internet Support Group and the participants’ employment status. No significant correlations were found between employment status and the variables of Internet Support Group use (Kruskal-Wallis test).

Group		Rank sum	*P* value
**Interaction (n)**			**.90**
	Employed/full or part time (15)	88.00	
	Pension/other (14)	329.50	
	Sick leave/unemployed (19)	507.50	
**Page views (n)**			**.80**
	Employed/full or part time (15)	388.00	
	Pension/other (14)	319.50	
	Sick leave/unemployed (19)	517.30	
**Posts (n)**			**.82**
	Employed/full or part time (15)	399.00	
	Pension/other (14)	344.00	
	Sick leave/unemployed (19)	481.00	
**Comments (n)**			**.95**
	Employed/full or part time (15)	370.00	
	Pension/other (14)	362.50	
	Sick leave/unemployed (19)	492.00	

**Table 4:** Correlation between activity on the Internet Support Group and marital status and gender (n=48).

Group		n (%)	Interaction		Page views		Posts		Comments	
			Rank sum	*P* value	Rank sum	*P* value	Rank sum	*P* value	Rank sum	*P* value
**Sex**				**.31**		**.35**		**.04^a^**		**.10**
	Male	22 (46)	475.5		478.5		442.5		451	
	Female	26 (54)	749.5		746.5		782.5		774	
**Marital status**				**.85**		**.87**		**.71**		**.26**
	Married/cohabitating	41 (85)	1032		1030		1036.5		1062.5	
	Living alone	7 (15)	193		194		188.5		162.5	

^a^Significant correlation was found between female gender and the contribution of posts on the Internet Support Group (Wilcoxon Rank-sum).

**Table 5:** The presence of anxiety and depression at baseline (n=48).

Anxiety/Depression	+Depression, n (%)	–Depression, n (%)	Total, n (%)
+Anxiety	8 (17)	6 (14)	14 (29)
–Anxiety	9 (19)	25 (52)	34 (73)
Total	17 (35)	31 (65)	48 (100)

**Table 6:** Use of Internet Support Group (ISG) in participants with or without anxiety and depression at baseline (n=48). No significant correlations were found between the presence of anxiety or depression and variables of ISG use (Kruskal-Wallis ranks test).

Group			n (%)	Rank sum	*P* value
**Interactions**					
	**Anxiety**				**.85**
		–	34 (73)	841.50	
		+	14 (29)	383.50	
	**Depression**				**.35**
		–	31 (65)	868.50	
		+	17 (35)	356.50	
**Page views**					
	**Anxiety**				**.67**
		–	34 (73)	830.50	
		+	14 (29)	394.50	
	**Depression**				**.17**
		–	31 (65)	888.00	
		+	17 (35)	337.00	
**Posts and comments**					
	**Anxiety**				**.07**
		–	34 (73)	773.50	
		+	14 (29)	451.50	
	**Depression**				**.59**
		–	31 (65)	848.00	
		+	17 (35)	377.00	

**Table 7:** Interactions and page views in groups who are passive users or active users (n=48).

Group		n (%)	Rank sum	*P* value
**Interactions**				**<.001^a^**
	Passive users	25 (52)	485.00	
	Active users	23 (48)	374.00	
**Page views**				**<.001^b^**
	Passive users	25 (52)	463.50	
	Active users	23 (48)	761.50	

^a^Significant correlation was found between passive users and interactions.

^b^Significant correlation was found between active users and page views.

Table 7 shows the analysis of user variables comparing passive users with active users. Those users who were active had the most page views and passive users had the most interactions (Table 7).

### Interaction on the Internet Support Group

The total number of interactions on the ISG was 3357 in a 2-year period from the first participant entered until the last included participant reached 6 months follow-up after surgery. The ISG contained 180 conversation threads, generating 354 comments. The 180 conversation threads in the ISG were constituted by 671 independent dialogue sequences. On the basis of those 671 dialogue sequences, 7 thematic categories emerged: social recognition, experience of pain or use of pain medication, experience of physical activity or physical rehabilitation, expression of psychosocial well-being, and advising on and exploring the ISG and employment. Examples of findings within the 7 categories are shown in Textbox 1, and a short description of each of the categories is provided below. The categories are presented starting with the one including the largest number of threads and dialogue sequences and ending with the one with the fewest.

**The 7 categories and their associated dialogue sequences extracted from dialogs among the 23 active users of the Internet Support Group, including the 6 former patients who had a facilitating role.**

Social recognition

- Dialogue sequence 3: “I would like to hear more from you, lets hook up on Facebook.”
- Dialogue sequence 6: “Now it is holiday for a lot of people...I hope for sun to shine.”
- Dialogue sequence 170: “I was wondering, where are you all from?”
- Dialogue sequence 175: “I would like to meet up with you, I will text you.”
- Dialogue sequence 209: “Today I have been visiting Eva, it was very nice. I am grateful that she wanted to meet up with me and share her experiences.”
- Dialogue sequence 120: “Hello and welcome to all new..”
- Dialogue sequence 303: “The best to you all...and have a nice weekend.”

Experience of pain or use of pain medication

- Dialogue sequence 308: “I have had a lot of pain since the operation...I guess that is what to expect.”
- Dialogue sequence 317: “I am in a lot of pain, but until now I have been able to keep it on a bearable level using medication and red wine at night. That doesn´t work anymore...now I can´t walk.”
- Dialogue sequence 332: “I decreased the medication to quickly...I have no patience.”
- Dialogue sequence 389: “...I do not use morphine anymore and my nausea is gone as well. I take paracetamol four times a day and that is enough.”

Experience of physical activity or rehabilitation

- Dialogue sequence 426: “Hello! Today I have been walking for an hour, but it was hard, I had to rest when I got home. We were walking quite calm, but I had to rest during the walk as well.”
- Dialogue sequence 454: “I am only able to do the exercises some days and I can´t do all of the exercises. I just started walking outside and it is eight weeks since I was operated.”
- Dialogue sequence 483: “Yes, you need to be careful and not pressure you self too much. I walked a bit more than one kilometre yesterday, it went okay. But when I came home my back hurt...I had gone too far.”
- Dialogue sequence 503: “I start rehabilitation next week, I am looking forward to it.”

Expression of psychosocial well-being

- Dialogue sequence 521: “I wept of pain and experienced the feeling of being small and miserable.”
- Dialogue sequence 524: “I do believe it will get better, I have always said that I would turn 107.”
- Dialogue sequence 531: “I want to hear how you are...I am going out of my mind”
- Dialogue sequence 534: “It is very difficult when I am used to being social and active, it almost makes me cry. I am almost eight weeks after surgery and now I think it is time it got better. I need more patience – I am looking forward to hearing from you all.”

Expression of everyday activities

- Dialogue sequence 565: “I have found a new rhythm for my day, I watch TV, I do crosswords and I relax, it is nice.”
- Dialogue sequence 575: “Can anyone suggest things I should prepare for when I get home.”
- Dialogue sequence 596: “to those of you who are past the operation...is it possible to bend forward to tie your shoes? Or how do you do?”
- Dialogue sequence 600: “any suggestions of how-to relief the back when cooking? I want to cook; however, I can´t stand by the kitchen table for so long.”

Advising on and exploring the Internet Support Group

- Dialogue sequence 608: “I have worked out how to get in (on the ISG) using my PC, so I am still here.”
- Dialogue sequence 618: “God morning everyone – I was thinking how do we get more activity here? I know that when all newly operated may not have the energy. But the rest of us? Any ideas?”
- Dialogue sequence 621: “I hope you all want to join; our experiences are worth gold for others.”
- Dialogue sequence 625: “Just write something about the weather, if you don´t want to share your health – that is okay. It is better to write something than nothing.”

Employment

- Dialogue sequence 634: “I do some voluntary work at a reception, I hope to start again this January.”
- Dialogue sequence 643: “I am now 3 months and 15 weeks and I just started working 4 hours each week...Yes, it is not much, but it is very important for me.”
- Dialogue sequence 647: “Nice to hear that you found a rhythm working, which suits your back – It is a fine line, which is hard to find.”
- Dialogue sequence 655: “When you get better I hope you will find a job than both you and your back can manage.”

### Social Recognition

The category represented the largest number of threads (n=145, 81%) and largest number of dialogue sequences (n=307, 46%) related to social recognition in and of the ISG. This category had 3 subthemes: The largest subtheme comprising a substantial number of the total dialogue sequences (n=233, 35%) was supportive comments, where group members welcomed new members to the ISG, expressed thinking of each other, and expressed concern and well-wishes for other group members. Another subtheme within social recognition was the invitation to other group members to interact outside the ISG. Finally, a number of dialogue sequences comprised more ordinary chitchat unrelated to the LSF surgery and recovery; they also comprised exchanging stories on how to spend their vacation, enjoying the spring or about serious subjects, such as losing a family member.

### Experience of Pain or Use of Pain Medication

The discussion of pain or pain medication was prevalent in 56 threads (31%), comprising 116 dialogue sequences (17%). Dialogues concerning pain medication either contained posts or comments on how to decrease the use of pain medication or expressions of what was found to be the side effect of the medication. Finally, expressions of either increase or decrease of pain after surgery was a recurrent topic of conversation.

### Experience of Physical Activity or Physical Rehabilitation

In 45 threads (25%) and 82 dialogue sequences (12%), posts or comments focused on the experience of walking or seeking to motivate each other to walk. Finding the balance between walking and resting was another recurrent line of conversation, as well as the start of active rehabilitation.

### Expression of Psychosocial Well-Being

Psychosocial well-being was a key focus in 34 threads (19%) of the discussion, appearing in 54 dialogue sequences (8%). These could be divided in 2 subthemes, 1 which was positive statements of future expectations, such as expressions of being optimistic of future recovery. The other was expressions of anxiety, lack of energy, or hard times.

### Expression of Everyday Activities

The request of information from peers was a key theme in the use of the ISG. A total of 24 threads (13%) contained 42 dialogue sequences (6%) concerning request of information. A total of 35 of these focused on expression of everyday situations, sharing with the groups their experience on how to manage cooking, pass time, etc. A total of 9 dialogue sequences focused on health-related information, such as how to tend to the bandages or the itching of the wound.

Advising on and Exploring the Internet Support Group

In 18 threads (10%), 28 dialogue sequences (4%) were passing advice among members on the use of the ISG. These dialogue sequences were a mixture of advice from one to the other of where to write what type of content, invitations to upload posts and to share stories, and dialogues on how to create more activity in the ISG.

### Employment

A common concern for many of the LSF patients who used the ISG was related to employment. This came up in 16 threads (9%), comprising 42 dialogue sequences (6%), for example, commenting on the number of hours of employment it was possible to undertake, the lack of employment, or the experience of being fired following the LSF surgery.

## Discussion

### Socially Constructed Patterns in Internet Support Group Usage

Gender distribution was almost equal in this study, with no significant differences between men and women with regard to the use of the ISG, which is in line with previous findings [35,45,46]. However, significantly, more women were active users, contributing by uploading posts. This is in line with other findings where women were found to be more than 4 times as likely to be active users, contributing with written uploads [47]. Explanations are offered for this discrepancy, focusing on socially-constructed patterns of behavior [46]. The general division of labor and the pay gap within and between occupations are generally responsible for larger incomes among men. Thus, it rationally makes more financial sense that the woman in a partnership takes time off to perform care duties, making them more acquainted with and prone to engage in health care information on the internet [46]. Another explanation proposed as to why men may be averse to asking questions and posting comments in an ISG is that they generally act according to a set of masculine values: asking questions requires a confession of ignorance or need, which may pose a threat to masculinity [46]. Furthermore, men generally tend to consider health situations to be less harmful, generating less motivation to ask questions and seek information from others [46]. Supporting this argument, the Comprehensive Model of Information Seeking points out that a person’s needs or perceptions of risk influence the degree to which the ability to do something about a health problem is considered to be realistic, thereby generating information seeking behavior [48]. That perceived risk influences social behavior is also found in another study published from the United Kingdom that included 863 depressed and anxious participants [24]. They found that anxious and depressed participants were more prone to take part in an ISG [24]. Participants with symptoms of anxiety in this study also tended toward being more prone to uploading posts and comments. Thus, the degree of contribution in this study seems to be gender related and could further be related to mental health state.

There was no sociodemographic variable in this study that correlated with the use of the ISG. Lack of such predictors was also found in the previous mentioned study from the United Kingdom [24]. However, others find that socioeconomic status, comprising education and employment status, correlates with ISG use [6,35,47,49]. Some of the explanation for the discrepancy among findings might be found in the results of a US study, with data drawn from 2358 participants [47]. In that study, a differentiation was made between seeking information and engaging in social media. It was concluded that lower socioeconomic status, older age, and male gender were associated with less likelihood to engage in eHealth activities, such as seeking information; it was also concluded that lower socioeconomic status was associated with a greater likelihood to use health-related social media [47]. In the context of this study, the ISG was hosted on a website with additional animated information. Thus, the website incorporated both information and social interaction, and it might appeal to a broader audience, limiting the possibility of finding predictors for ISG use.

### Provision of Information and the Nature of Posts

Provision of animated information on the website might have had further influence on the ISG use. We chose not to extract dialogue sequences categorized as seeking of information, as the request of information or giving recommendations did not dominant the correspondence. Posts and comments were to a larger extent characterized by the expression of sharing experience, supporting each other and engaging in common dialogues. In a recently published study, done on bariatric surgery support groups and pages on Facebook, 11% of the content indicated seeking of information and 53% of the content contained the provision of information and recommendations [50]. The additional animated information might have reduced the need for information from peers.

Furthermore, post hoc analysis found no posts or comments containing incorrect or contradictive information in this study. Using ISGs, patients are found to be concerned about the quality of information being shared, and they occasionally encounter contradictive information, actually adding to patient’s insecurity rather than the opposite [5]. The animated information provided on the website, together with the ISG, may have reduced some of these misconceptions. Thus, the provision of an ISG on a website, together with information, might appeal to a larger audience and might also reduce the sharing of misinformation. In addition, the ISG was used in the context of a research study, where the participants were being monitored, which also might have made them reluctant to exchange information.

### Social Recognition

Social recognition was the largest thematic category emerging from the posts and comments, with a substantial number of dialogue sequences (n=307, 46%), and with supportive posts and comments being the largest subtheme with 233 (35%) dialogue sequences. A possible explanation for the many supportive comments and posts might be found in the characteristics of the patient group. There seems to be a distinction between which condition the participant has and the nature of posts and comments made. Mental health disease ISGs comprise more emotional support, although it seems that the requests for information relate more toward physical conditions [51]. LSF is performed because of a physical condition; however, as presented above, half of the participants had symptoms of anxiety or depression, and 16% had symptoms of both, which might contribute to the large amount of supportive posts. Furthermore, the 7 thematic categories emerging from the content analysis, social recognition, experience of pain or use of pain medication, experience of physical activity or physical rehabilitation, expression of psychosocial well-being, advising on and exploring the ISG and employment, correspond to a large extent to the factors, which have been found to be associated with symptoms of anxiety and depression within patients undergoing spinal surgery, such as pain, information, disability, employment, and mental health [52]. Thus, the often occurrence and nature of supportive posts might be because of the prevalent occurrence of symptoms of anxiety or depression, which have been found to accompany one-third of spine surgery patients [25-28].

### Implementation of the Internet Support Group

A greater percentage of participants accessed the ISG in this study than in other studies evaluating a wider use of ISGs and in participants with other diagnosis [24,35]. There may be several reasons for this degree of participation. A total of 3 possible explanations will be explored below. First, a choice was made to increase accessibility, and patients were offered the use of a tablet. This choice may have increased the participants’ perceived ease of use and accessibility of the ISG, which may have facilitated a more positive attitude and frequency of use. That ease of use influences acceptance and adoption is in concordance with the theory of the Technology Acceptance Model [53] and the previously mentioned implementation theory [39]. Second, all participants in this study agreed to participate, knowing that they would have to relate to an ISG. Agreeing to these terms may have created more motivated users. A third possible explanation could be related to the ISG being embedded on a website, with an additional number of separate components available as well. A broader assortment of support may be appealing and useful for a wide range of participants, attracting both those seeking information and those seeking health-related social media. The influence of perceived usefulness is in accordance with the Technology Acceptance Model and implementation theories, and it is found to support a positive attitude and a behavioral intention toward use of a new technology [39,53]. Thus, in the light of the above the choice to increase accessibility, the motivation and the perceived usefulness might have been facilitators, increasing the use of the ISG in this study.

A small number of the participants in this study were responsible for the majority of interactions. The 3 most active users contributed 25% of the total number of interactions. This phenomenon that a few contribute a lot is known as the peer leader phenomenon, characterized by a high posting frequency. This tendency is found to the extent that 1% of participants are seen to contribute 75% of all posts [54]. Such peer leaders identify themselves as active help providers, tending to provide a high level of social support, which contributes to an increased effect of the ISG if social support underpins the improvement [54]. There is a high amount of supportive comments comprising 35% of all extracted dialogue sequences in this study. This provides some indication of the nature of the social behavior in the ISG employed in this study, as there seems to be a positive tone among the participants.

Approximately half of the participants in this study (48%) contributed by posting or making comments. It is known that members of online groups often begin their membership as visitors or so-called lurkers, who just observe, and that not all members shift from being visitors to active users [55]. Some retain a passive behavior, as members are mainly interested in the information they can access through the online group [55]. In this study, the passive users are those with the most page views, which may indicate that they were interested in the information they could retrieve. It is possible that the fact that interactions within ISGs comprise mainly of text has an impact on patterns of use to the extent that only half of the users were active and contributors in this study. This type of Web-based interaction does require that participants are comfortable writing and reading information on the Web. eHealth literacy (defined as the ability to seek, find, understand, and appraise health information from electronic sources and apply knowledge gained to address or solve a health problem) [56] is known to correlate with social position, older age, and chronicity of disease [47], and it may be one of the reasons why some of the participants in this study did not contribute actively to the ISG. It is suggested that education might be a more salient proxy for ISG use than income, indicating that determinants of eHealth literacy may be important predictors [47]. It was not possible to find correlations in this study confirming the level of education as a predictive factor for ISG use, the reason for this might be found in the high percentage of participants (69%) with a secondary education. However, it seems plausible that eHealth literacy must be taken into consideration when developing internet interventions. Failure to do so could increase the already existing inequality of health care. Further research should be done accessing eHealth literacy and its influence on eHealth engagement across social groups.

In the literature, expressed limitations of ISG use can also be found. Even though it is acknowledged that the key reason for citizens to participate in an ISG is the connection with others, a Web-based survey suggests that the lack of actual physical proximity makes relationships developed within an ISG less meaningful and actually makes patients feel even more isolated outside of the ISG [5]. Looking at the content of posts and comments, a subtheme did reveal invitation to other group members to interact outside the ISG.

### Limitations

This study has several imitations. First of all, the use of google analytics, together with user data and personal data, to uncover activity on the ISG has limitations. The quality of data could have been increased by the use of a unique user ID added to the platform’s user accounts. This could have automatically identified behavior on the ISG, providing more accurate data and a clearer picture of events than that provided in our manually generated process.

The number of participants is small, limiting the strength of the analysis and making it difficult to draw statistically sound conclusions. However, our sample does not deviate markedly from those in the Danish population who report having a spinal condition in relation to gender, age, educational level, and employment status [33]. Moreover, the prevalence of both anxiety and depression in this sample of LSF patients is equal to that found in the literature among a similar group of patients [25,27,28,57]. However, further research with a larger group of participants should be done, and it might uncover further knowledge on the characteristics of use of an ISG in patients undergoing LSF.

All participants not owning a tablet were offered to borrow one, and thus half of the participants (59%) chose to borrow such a device, clearly influencing the use of the ISG and the generalizability of the study results, as we are not able to conclude on the use of the ISG in a home without the use of a tablet.

All the participants did not access the ISG at the same time; the recruitment process was consecutive and slow in periods. It is not possible to know what influence this had on the frequency of use. To accommodate the slow recruitment process, 6 former patients were invited to help engage participants. The role of these 6 patients is not further uncovered within this study; however, their role as moderators may have had an influence on the usage, the content of uploaded posts and comments, and the quality of the study. Furthermore, all participants were aware that their interactions were being studied, and thus some might be more reluctant to engage in the ISG; this could be uncovered in future studies.

Activity on the ISG is seen as a positive contribution; however, the perceived value of the different contributions was not considered. We did look at the content of posts and comments that contributed to the knowledge of behavior within an ISG; however, an exploration of how the posts and comments were received could have been relevant and would provide additional knowledge on behavior in an ISG.

The full potential of internet use within health care has not yet been reached, for example, with regard to dissemination of information, establishing health community networks, blogging, and establishing support groups. It is important to acknowledge that this global availability of information and support comes with difficulties. We need to strive to accommodate these difficulties by providing high-quality eHealth accessible to all.

### Conclusions

This paper contributes to the literature on the use of ISGs within health care and especially within the group of patients undergoing LSF. Socioeconomic status was not an important barrier in this study, and it was not possible to find characteristic determinants for the use of ISG among this group of patients; however, the high use of an ISG in this study may confirm that an ISG is relevant for patients undergoing LSF. Socially constructed patterns of behavior may make women more prone to be contributors, and the perceived risk of spinal surgery experienced by participants with symptoms of anxiety may likewise make them more prone to ask questions and contribute actively. A total of 7 thematic categories emerged when exploring the content of posts and comments on the ISG: social recognition, experience of pain or use of pain medication, experience of physical activity or physical rehabilitation, expression of psychosocial well-being, and advising on and exploring the ISG and employment. The nature of posts and the thematic categories seem to correspond well to the prevalent occurrence of symptoms of anxiety and depression within the group of participants. Future studies need to focus on the perceived value of the ISG in LSF patients and on the usage behavior in an ISG when incorporating other features, such as animated information or instructions on a joint website.

## Abbreviations

eHealth: electronic health
HADS: Hospital Anxiety and Depression Scale
ISG: Internet Support Group
LSF: lumbar spine fusion
RCT: randomized controlled trial
